# Redox regulation of EGFR activation by thioredoxin reductase 3 drives resistance to EGFR inhibitors in triple-negative breast cancer

**DOI:** 10.1038/s41420-026-03157-0

**Published:** 2026-05-19

**Authors:** Prahlad V. Raninga, Göknur Giner, Sivanandhini Sankarasubramanian, Murugan Kalimutho, Marco J. Herold, Antoine de Weck, Kum Kum Khanna

**Affiliations:** 1 Mater Research Institute, The University of Queensland, Translational Research Institute, Woolloongabba, QLD Australia; 2 QIMR Berghofer Medical Research Institute, Brisbane, QLD Australia; 3 Walter and Eliza Hall Institute of Medical Research, Melbourne, VIC Australia; 4 Olivia Newton-John Cancer Research Institute, Heidelberg, VIC Australia; 5 School of Cancer Medicine, La Trobe University, Bundoora, VIC Australia; 6 Department of Medical Biology, University of Melbourne, Parkville, VIC Australia; 7 Children’s Cancer Institute, Sydney, NSW Australia

**Keywords:** Breast cancer, Cancer therapy

## Abstract

Although 40–70% of TNBC cases overexpress EGFR, clinical responses to EGFR-targeted therapies have been minimal. This poor efficacy may result from intrinsic resistance mechanisms, inactive EGFR signaling, or reduced EGFR localization on the plasma membrane. To identify genetic determinants of EGFR inhibitor resistance, we performed a genome-wide CRISPR/Cas9 knockout screen in MDA-MB-231 cells. The screen revealed that loss of the redox-regulating enzyme Thioredoxin Reductase 3 (TXNRD3) sensitized TNBC cells to the EGFR inhibitor erlotinib. Functional validation showed that both siRNA-induced knockdown and pharmacological inhibition of TXNRD3 with the FDA-approved drug auranofin significantly enhanced the cytotoxic effects of EGFR inhibitors in EGFR-high TNBC cells. Mechanistically, TXNRD3 depletion or inhibition increased intracellular reactive oxygen species (ROS), leading to oxidation-dependent activation and phosphorylation of EGFR (Y1068) and subsequent activation of downstream signaling pathways in TNBC cells that otherwise lack active EGFR. The combined treatment of auranofin and EGFR inhibitors triggered GSDME-mediated pyroptosis in a ROS-dependent manner. Importantly, the combination of auranofin with erlotinib exhibited potent anti-tumor efficacy in vivo in both MDA-MB-231 xenograft and 4T1.2 syngeneic TNBC models. Collectively, our findings identify TXNRD3 as a redox-dependent regulator of EGFR activity and drug response in TNBC and demonstrate that auranofin-mediated TXNRD3 inhibition can re-activate EGFR signaling, thereby sensitizing TNBC tumors to EGFR-targeted therapy. This study provides a mechanistic rationale for repurposing auranofin in combination with EGFR inhibitors as a novel therapeutic strategy for EGFR-high TNBCs.

## Background

Triple-negative breast cancers (TNBCs) are the most aggressive subtype of breast cancer, accounting for approximately 15–20% of all diagnosed invasive breast cancer cases worldwide and are more prevalent amongst younger women ( < 40 years of age). TNBCs are highly heterogeneous and can be subdivided into four types, including basal-like, luminal, mesenchymal, and immune-modulatory/activated [1]. Due to the lack of surface hormone receptors and HER2 amplification, TNBCs do not respond to anti-hormonal or HER2-directed therapies, unlike luminal and HER2+ breast cancers. Hence, chemotherapies and radiotherapy remain the mainstay of treatment for TNBC patients. Although many TNBC patients initially respond to chemotherapies, these tumors frequently relapse and metastasize, particularly to the lungs and brain, and acquire chemoresistance [2]. Unfortunately, the severe adverse effects and drug resistance associated with standard cytotoxic chemotherapies (e.g. anthracyclines, cyclophosphamide, and taxanes) significantly limit their long-term clinical benefits in TNBC patients [3, 4]. Thus, there is an urgent need to explore novel, clinically viable targeted therapies for primary and metastatic TNBCs.

Several studies have reported EGFR overexpression in 40-70% of TNBC patients by immunohistochemistry [5, 6]. EGFR overexpression correlates with poor clinical outcome and decreased disease-free survival in TNBC patients [5]. Elevated EGFR messenger RNA (mRNA) expression and pathway activation are characteristic of basal-like breast cancer [7, 8] and are associated with adverse outcomes [9]. Despite this, a clinical trial of a small molecule EGFR inhibitor, Erlotinib, as monotherapy or combination in unselected previously treated women with locally advanced or metastatic breast cancer, metastatic and recurrent TNBC showed minimal efficacy [10]. Similarly, the phase II trial of an EGFR monoclonal antibody, cetuximab, in combination with carboplatin in stage IV TNBC patients showed no significant clinical response [11], suggesting the existence of intrinsic resistance mechanisms. Understanding and overcoming these resistance pathways could enhance the therapeutic efficacy of EGFR-targeted agents.

In this study, we performed a genome-wide CRISPR/Cas9 knockout screening in TNBC cells treated with erlotinib to identify genetic drivers of erlotinib resistance. Our analysis revealed that thioredoxin reductase 3 (*TXNRD3)*, a member of the thioredoxin protein family involved in maintaining intracellular redox homeostasis, is a critical driver of erlotinib resistance in TNBC cells. Genetic depletion of *TXNRD3* markedly sensitized TNBC cells to EGFR inhibitors, including erlotinib and osimertinib. Supporting this finding, previous studies have reported that NRF2-driven antioxidant genes, including TXNRDs, are upregulated in osimertinib-tolerant lung cancer drug persister cells, implicating the thioredoxin pathway in EGFR inhibitor resistance [12]. Notably, pharmacological inhibition of TXNRD3 using the FDA-approved thioredoxin inhibitor auranofin synergized with EGFR inhibitors to suppress basal-like TNBC cell growth in vitro and in vivo.

Together, these findings identify TXNRD3 as a novel regulator of EGFR inhibitor sensitivity in TNBC and suggest that combined targeting of EGFR and thioredoxin pathways represents a promising therapeutic strategy for basal-like TNBCs.

## Results

### Genome-wide CRISPR/Cas9 screen identifies *TXNRD3* as a critical mediator of EGFR inhibitor resistance in TNBC cells

**Fig. 1:**
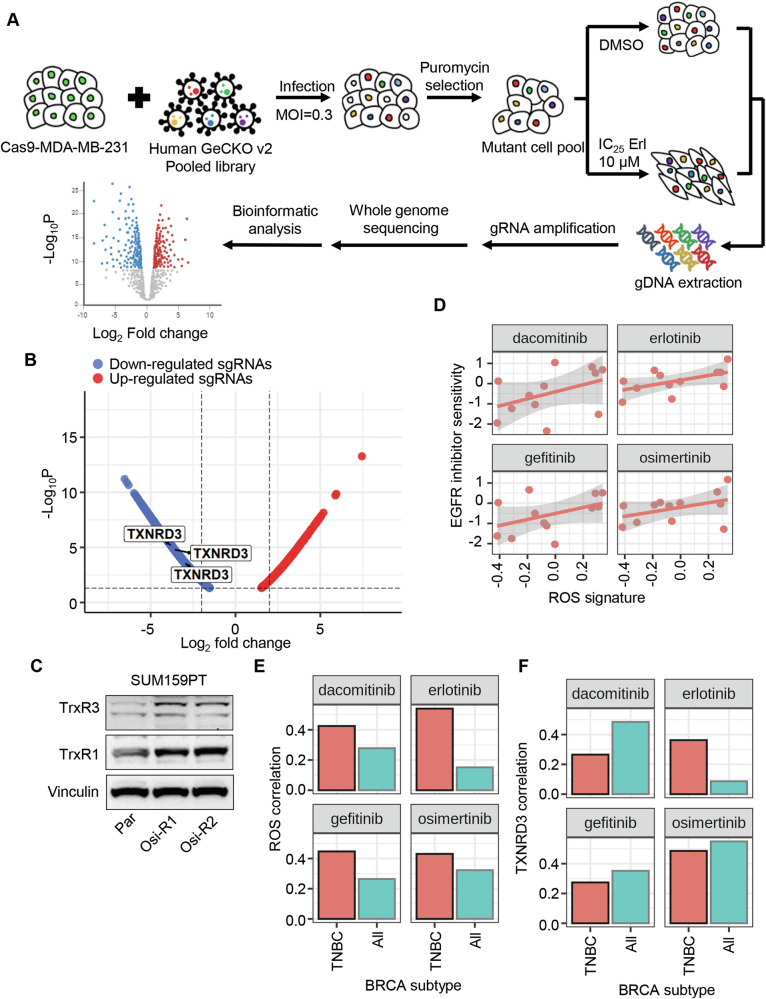
CRISPR library screening identified *TXNRD3* as a critical gene for erlotinib resistance in TNBC cells. **A** Schematic diagram illustrating the workflow of genome-wide CRISPR/Cas9 knockout library screening (CRISPR: Clustered Regularly Interspaced Short Palindromic Repeats) of Cas9-MDA-MB-231 cells treated with either DMSO or erlotinib (IC_25_, 10 µM). The Fig. was adapted [35] and modified using BioRender. **B** Volcano plot illustrating the differential guides significantly depleted (in blue) or enriched (in red) in erlotinib-treated MDA-MB-231 cells compared to DMSO-treated control MDA-MB-231 cells. The differential guides were plotted with statistical significance (-log_10_ *p*-value) on the y-axis against fold change (log_2_) on the x-axis. Each point represents a guide, with those above the significance threshold with a fold change greater than 2. TXNRD3 guides are highlighted. Source data are provided as a supplementary source file. **C** Protein levels of thioredoxin reductase 1 (TrxR1) and thioredoxin reductase 3 (TrxR3) were analyzed by Western blot analysis in parental and osimertinib-resistant or persister SUM159PT cells. Representative images of three independent Western blot analysis are shown. Vinculin was used as a loading control. **D** Linear regression (solid line) of EGFR Sensitivity values (circles) on ROS signature among the 12 TNBC cell lines in CCLE. The shaded region is 95% confidence interval of the linear regression. The regression slopes are consistently positive - Dacomitinib: 1.75, Erlotinib: 1.17, Gefitinib: 1.49, and Osimertinib: 1.12. **E** Pearson correlation coefficient between ROS signature and Sensitivity of EGFR inhibitors across TNBC only (red) or all breast cancer cell lines (blue). **F** Pearson correlation between EGFR sensitivity and TXNRD3 expression across TNBC (red) and all breast cancer cell lines (blue).

To identify genes conferring resistance to the EGFR inhibitor, we performed a genome-wide CRISPR/Cas9 knockout screen in human TNBC cells. Cas9-expressing MDA-MB-231 cells, which exhibit high EGFR expression, were transduced with human GeCKO v2 CRISPR lentiviral library (contains 123,411 unique sgRNAs, targeting 19,052 protein-coding genes, 1864 microRNAs, and 1000 control sgRNAs) at an MOI of 0.3 (Fig. 1A). Following puromycin selection, >90% sgRNA retention and ~400× library coverage was achieved (Fig. S1A), ensuring sufficient read depth and library coverage for the screen. Cells were treated with vehicle or erlotinib (10 µM; IC₂₅) for 7 days, and sgRNA abundance was quantified by next-generation sequencing. We removed non-targeting sgRNAs and those with low expression to improve data quality and determined the library sizes in each treatment condition (Fig. S1B).

We hypothesized that knockout of erlotinib-resistance driver genes would sensitize TNBC cells to erlotinib-induced cell death or growth inhibition. Analysis of depleted sgRNAs revealed 1984 genes significantly reduced (*P* < 0.05) in erlotinib-treated cells relative to controls (Fig. 1B h& Additional file 1). Among these, 88% of genes were represented by ≥4 independent sgRNAs (Fig. S1C). For validation, we prioritized the top 150 significantly depleted genes as high-confidence candidates mediating resistance to erlotinib. Many of these genes are transcription factors, zinc-finger proteins, small uncharacterized proteins, or mitochondrial/ribosomal components with no specific inhibitors and are not currently druggable.

Among these 150 genes, 11 were found to have existing FDA-approved inhibitors targeting either the gene itself or its functional pathway. Several of these genes, such as ABCG2, STAT1, and PSEN1, have been previously implicated in mediating EGFR inhibitor resistance across diverse cancers. Overexpression of ABCG2, a multidrug efflux transporter, confers resistance to EGFR inhibitors by reducing their intracellular accumulation, thereby diminishing their cytotoxic efficacy [13, 14]. STAT1, a downstream effector of JAK signaling, has been linked with EGFR inhibitor resistance in KRAS mutant colon cancer cells [15]. PSEN1, a core component of the γ-secretase complex, drives EGFR inhibitor resistance by regulating EGFR expression and activation in colorectal cancer [16].

In contrast, a subset of genes, including TXNRD3, CHKA, and IGFBP6, possess FDA-approved pharmacological inhibitors but have not previously been linked to EGFR inhibitor resistance, suggesting novel therapeutic vulnerabilities. Notably, TXNRD3 emerged as a high-confidence depleted gene upon erlotinib treatment, with all three independent sgRNAs targeting TXNRD3 consistently and significantly lost following drug exposure (Fig. 1B). *TXNRD3* is a member of the thioredoxin system. Three thioredoxin reductase isoforms are found in mammals, and are selenocysteine-containing flavoenzymes, which reduce thioredoxins and other cysteine-containing proteins, and play a key role in redox homeostasis [17]. *TXNRD3* localizes to mitochondria and, unlike the other two isoforms, contains an additional N-terminal glutaredoxin domain that enables *TXNRD3* to participate in both thioredoxin and glutathione antioxidant systems. The GeCKO v2 library includes sgRNAs targeting all three thioredoxin reductase paralogs (TXNRD1, TXNRD2, and TXNRD3), enabling assessment of potential functional redundancy within this enzyme family. Notably, TXNRD1 showed a much weaker signal in the screen, ranking 10,377th with a smaller effect size (logFC = −7.3, *p* = 0.029), and therefore did not meet our predefined criteria for high-confidence hits. TXNRD2 did not show significant depletion or enrichment under erlotinib treatment conditions. These data suggested a degree of specificity for TXNRD3 within the thioredoxin reductase family in the context of EGFR inhibitor resistance.

Importantly, auranofin, an FDA-approved gold-containing compound originally used for rheumatoid arthritis, is a known inhibitor of thioredoxin reductases, including TXNRD3 [17]. Given the critical role of redox balance in cell survival under EGFR inhibition, these findings highlight TXNRD3 as a highly druggable and mechanistically relevant target whose inhibition could sensitize resistant TNBC cells to erlotinib. Thus, among the top resistance drivers identified, TXNRD3 stands out as a clinically actionable target with immediate translational potential for combination therapy with EGFR inhibitors.

### TXNRD3 expression is upregulated in EGFR inhibitor–resistant TNBC cells and correlates with poor patient outcomes

We next assessed *TXNRD3* protein levels in osimertinib-resistant TNBC cells. SUM159PT cells, a TNBC cell line with high EGFR expression, were treated with osimertinib (IC₉₀) for 14 days to enrich for drug-persistent cells. Immunoblotting revealed a significant upregulation of TXNRD3 protein in osimertinib-persistent versus parental (untreated) cells (Fig. 1C). Consistent with our observations, TXNRD3 has been shown to be upregulated in TNBC cell lines and tumor tissues and is associated with adverse prognosis in TNBC patients [18]. Moreover, higher TXNRD3 expression has been reported in sorafenib-resistant leukemia and hepatocellular carcinomas [17]. High TXNRD3 activity maintains thioredoxin 2 in a reduced state, which stabilizes several anti-apoptotic proteins, including Bcl-XL, Bcl-2, and MCL-1, thereby promoting drug resistance [17], further suggesting the role of *TXNRD3* in conferring drug resistance. Notably, although TXNRD3 is expressed at lower basal levels relative to TXNRD1, its selective induction in drug-persistent cells suggests that TXNRD3 expression is dynamically regulated in response to EGFR inhibitor pressure rather than being determined by absolute abundance. Hence, its selective depletion in the CRISPR screen and induction in drug-persistent cells suggest that context-dependent upregulation, rather than absolute baseline abundance, underlies its role in EGFR inhibitor resistance.

We next tested whether TXNRD3 expression or ROS gene signature [12] correlates with EGFR inhibitor sensitivity across breast cancer cell lines using the publicly available PRISM Drug Repurposing dataset. The resulting combined data included sensitivity to 4 EGFR inhibitors (dacomitinib, erlotinib, gefitinib, and osimertinib), the transcript per million counts for TXNRD3, and a gene signature value for ROS across 26 breast cancer cell lines from the CCLE, of which 12 are TNBC. Comparing ROS signature and sensitivity to EGFR inhibition across TNBC cell lines, we observed a consistently positive correlation (Fig. 1D, E) (*p*-value < 0.05 with Brown’s method aggregation across 4 EGFRi), whereby sensitive cell lines have a lower ROS signature than refractory lines. When expanding to all breast cancer cell lines, the correlation is lower but remains positive (Fig. 1E). Furthermore, we also observed a positive correlation with high TXNRD3 mRNA levels and EGFR inhibitor sensitivity; TNBC cells with TXNRD3 expression were resistant to EGFR inhibitors (Fig. 1F). Together, this provides evidence linking ROS and TXNRD3 to EGFR resistance in TNBC and suggests an early stratification strategy.

Furthermore, Kaplan–Meier (KM-Plotter) analysis of TNBC publicly available datasets [19] demonstrated that high *TXNRD3* expression was associated with significantly shorter relapse-free and overall survival (Fig. S2A). Additionally, *TXNRD3* levels (probe 221906_at) were elevated in chemotherapy non-responders relative to responders (Fig. S2B), with ROC analysis yielding an AUC of 0.60 (*p* = 0.012) [20]. These data indicate that *TXNRD3* upregulation correlates with treatment resistance and poor prognosis in TNBC patients.

### Genetic depletion of TXNRD3 sensitized TNBC cells to EGFR inhibitors

**Fig. 2:**
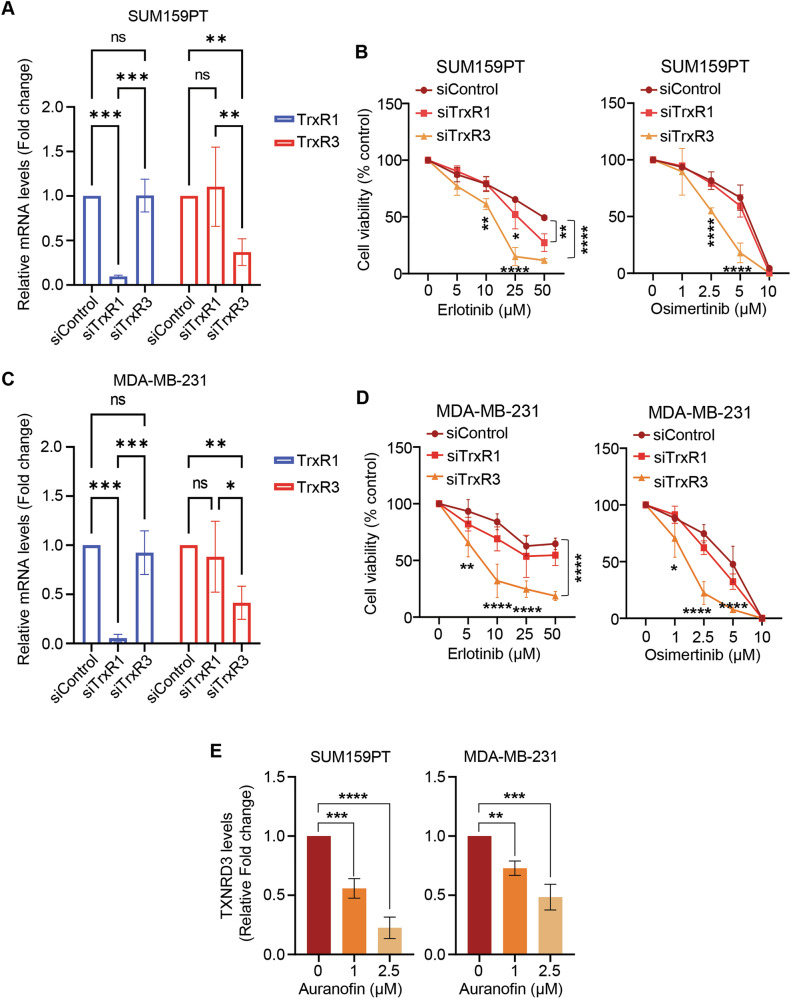
Genetic depletion of TXNRD3 sensitized TNBC cells to EGFR inhibitors. **A** SUM159PT cells were transfected either with scramble or control siRNA, TrxR1-specific siRNA, or TrxR3-specific siRNA for 48 h. TrxR1 and TrxR3 mRNA levels were analyzed by RT-qPCR analysis. One-way ANOVA followed by Tukey’s post-test, *n* = 3 independent biological replicates (mean ± SEM). **B** SUM159PT cells were transfected with either scramble or control siRNAs, TrxR1-specific siRNAs, or TrxR3-specific siRNAs for 24 h and subsequently treated with erlotinib (0-50 µM) or osimertinib (0-10 µM) for 72 h. Cell viability was analyzed by MT cell viability assays. One-way ANOVA followed by Tukey’s post-test, *n* = 3 independent biological replicates (mean ± SEM). **C** MDA-MB-231 cells were transfected either with scramble or control siRNA, TrxR1-specific siRNA, or TrxR3-specific siRNA for 48 h. TrxR1 and TrxR3 mRNA levels were analyzed by RT-qPCR analysis. One-way ANOVA followed by Tukey’s post-test, *n* = 3 independent biological replicates (mean ± SEM). **D** MDA-MB-231 cells were transfected with either scramble (control) siRNAs, TrxR1-specific siRNAs, or TrxR3-specific siRNAs for 24 h and subsequently treated with erlotinib (0-50 µM) or osimertinib (0-10 µM) for 72 h. Cell viability was analyzed by MT cell viability assays. One-way ANOVA followed by Tukey’s post-test, *n* = 3 independent biological replicates (mean ± SEM). **E** SUM159PT and MDA-MB-231 cells were treated with auranofin (0-2.5 µM) for 24 h. Intracellular levels of TXNRD3 were analyzed by ELISA assays. One-way ANOVA followed by Tukey’s post-test, *n* = 3 independent biological replicates (mean ± SEM).

To test whether TXNRD3 directly mediates resistance, we silenced *TXNRD3* or *TXNRD1* (the cytosolic isoform) using siRNAs in SUM159PT and MDA-MB-231 cells (Fig. 2A, C). TXNRD1 has been shown to be upregulated in TNBC cells [21]. Knockdown efficiency of TXNRD1 and TXNRD3 was confirmed by RT-qPCR (Fig. 2A, C). These knocked-down cells were treated with erlotinib (0–10 µM) or osimertinib (0–2.5 µM) for 72 h, and viability was measured via MTS assays. TXNRD3 knockdown markedly enhanced sensitivity to both EGFR inhibitors in both TNBC lines, whereas TXNRD1 knockdown had no effect (Fig. 2B, D). These results establish TXNRD3 as a specific mediator of EGFR inhibitor resistance in TNBC cells.

### Pharmacological inhibition of TXNRD3 using Auranofin sensitized TNBC cells to EGFR inhibitors in vitro

**Fig. 3:**
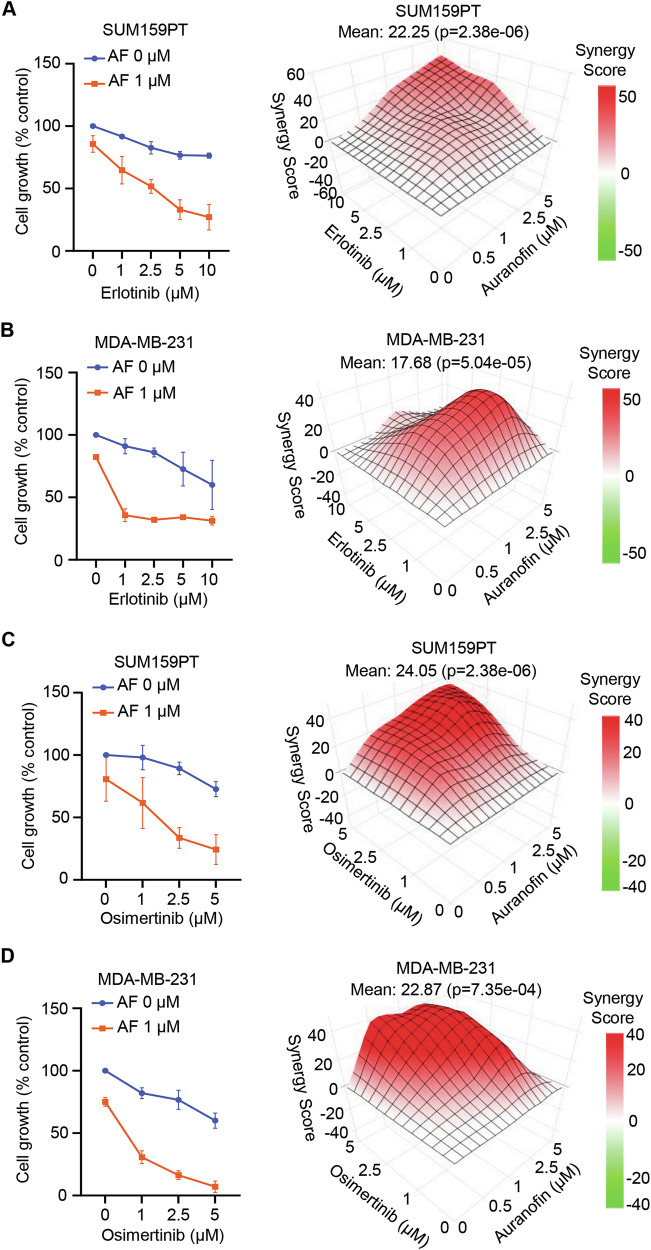
Pharmacological inhibition of TXNRD3 using auranofin sensitized TNBC cells to EGFR inhibitors in vitro. SUM159PT (**A**) and MDA-MB-231 (**B**) cells were treated with auranofin (AF) (0-5 µM) and erlotinib (0-50 µM), both alone and in combination, for 72 h, and cell viability was analyzed by MT cell viability assays. For cell growth curves (left panel), results with only 1 µM AF are shown. Synergy score was calculated using Synergy Finder. One-way ANOVA followed by Tukey’s post-test, *n* = 3 independent biological replicates (mean ± SEM). SUM159PT (**C**) and MDA-MB-231 (**D**) cells were treated with auranofin (0-5 µM) and osimertinib (0-10 µM), both alone and in combination, for 72 h, and cell viability was analyzed by MT cell viability assays. For cell growth curves (left panel), results with only 1 µM AF are shown. Synergy score was calculated using Synergy Finder. One-way ANOVA followed by Tukey’s post-test, *n* = 3 independent biological replicates (mean ± SEM).

**Fig. 4:**
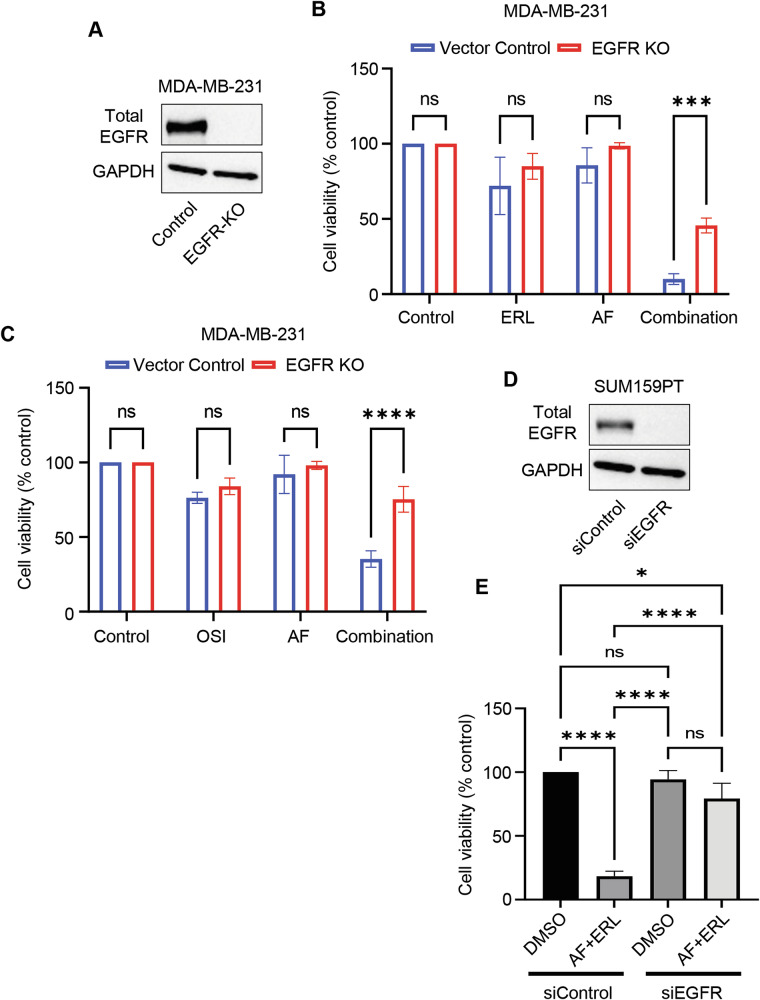
EGFR depletion rescues EGFR-high breast cancer cells from undergoing cell death upon auranofin-EGFR inhibitor combination treatment. **A** Western blot confirming EGFR depletion in CRISPR/Cas9 EGFR KO MDA-MB-231 cells compared to the CRISPR/Cas9 control MDA-MB-231 cells. Representative images of three independent experiments are shown (*n* = 3 independent biological replicates). **B** CRISPR/Cas9 control and EGFR KO MDA-MB-231 cells were treated with auranofin (2.5 µM), erlotinib (10 µM), either alone or in combination for 72 h. Cell viability was analyzed by MT Cell viability assays. One-way ANOVA followed by Tukey’s post-test, *n* = 3 independent biological replicates (mean ± SEM). **C** CRISPR/Cas9 control and EGFR KO MDA-MB-231 cells were treated with auranofin (2.5 µM), osimertinib (5 µM), either alone or in combination for 72 h. Cell viability was analyzed by MT Cell viability assays. One-way ANOVA followed by Tukey’s post-test, *n* = 3 independent biological replicates (mean ± SEM). **D** SUM159PT cells were transfected either with scramble siRNAs (siControl) or EGFR-specific siRNAs for 48 h. EGFR protein levels were analyzed by Western blot analysis. Representative images of three independent experiments are shown (*n* = 3 independent biological replicates). **E** SUM159PT cells transfected with either scramble siRNAs or EGFR-specific siRNAs were treated with auranofin (2.5 µM), erlotinib (10 µM), either alone or in combination for 72 h. Cell viability was analyzed by Crystal Violet cell viability assays. One-way ANOVA followed by Tukey’s post-test, *n* = 3 independent biological replicates (mean ± SEM).

Since genetic depletion of TXNRD3 sensitized TNBC cells to EGFR inhibitors, we next examined whether pharmacological inhibition of TXNRD3 using an FDA-approved thioredoxin reductase inhibitor, auranofin, could recapitulate this effect. Notably, auranofin has been shown to sensitize hepatocellular carcinoma cells to sorafenib, a pan-receptor tyrosine kinase inhibitor (PDGFR, VEGFR, c-Kit, and Ret), through TXNRD3 inhibition [17]. Auranofin treatment (0–2.5 µM, 24 h) reduced intracellular TXNRD3 levels in a dose-dependent manner (Fig. 2E). Next, we treated four EGFR-high TNBC cell lines, SUM159PT, MDA-MB-231, HCC1806, and HCC1143 (Fig. S1D), with a sub-lethal concentration of auranofin (1 µM) in combination with EGFR inhibitors (erlotinib and osimertinib) and analyzed cell viability using MT assays. In line with previous studies, both erlotinib and osimertinib as single agents had a minimal effect on TNBC cell growth (Figs. 3A–D and S3A–D). However, combination treatment with auranofin (1 µM) and erlotinib or osimertinib significantly reduced cell viability across four EGFR-high TNBC lines (SUM159PT, MDA-MB-231, HCC1806, and HCC1143) (Figs. 3A–D and S3A–D). Furthermore, we examined whether auranofin exerts synergistic anti-cancer activity with EGFR inhibitors using the BLISS synergy score, where a score >10 indicates synergy, a score < -10 indicates antagonism, and a score between –10 and 10 indicates an additive effect. BLISS synergy analysis indicated robust synergism (BLISS score >10) between auranofin and both EGFR inhibitors in all four TNBC cell lines tested (Figs. 3A–D and S3A–D). Importantly, this combination did not affect EGFR-low breast cancer lines (MCF7, BT-474, SKBR3; Figs. S1D and S4) and failed to sensitize EGFR knockout MDA-MB-231 or EGFR-silenced SUM159PT cells (Fig. 4B–E), confirming EGFR dependency.

Since auranofin inhibits both TXNRD1 and TXNRD3 [17, 21], we tested the TXNRD1 -selective inhibitor TRi-1 [22, 23]. Although TRi-1 effectively inhibited TXNRD1 redox activity (Fig. S5A), it did not sensitize TNBC cells to erlotinib (Fig. S5B), indicating that auranofin-mediated sensitization is unlikely to occur through TXNRD1 inhibition. Furthermore, pharmacologic inhibition of the glutathione antioxidant system using BSO did not enhance erlotinib sensitivity (Fig. S5C), suggesting that bulk glutathione depletion alone is insufficient to recapitulate the effects of TXNRD3 inhibition. Together, these findings support a TXNRD3-specific mechanism in EGFR inhibitor resistance, likely involving redox regulation beyond global glutathione metabolism.

### Auranofin and EGFR inhibitor combination treatment induces ROS-dependent, GSDME-mediated pyroptosis in TNBC cells

**Fig. 5:**
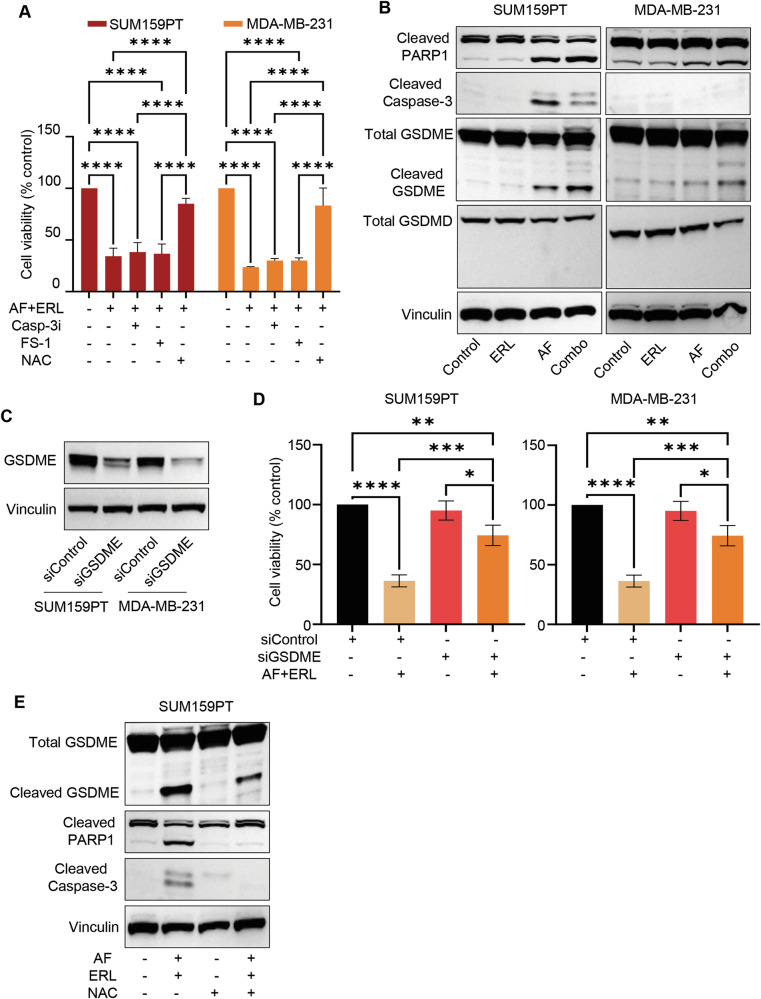
Auranofin and EGFR inhibitor combination treatment induces pyroptosis in TNBC cells. **A** SUM159PT and MDA-MB-231 cells were pre-treated with either pan-caspase inhibitor (Q-VAD-FMK, 20 µM), ferroptosis inhibitor (Ferrostatin, 50 µM), or ROS scavenger (NAC, 5 mM) for 3 h followed by the treatment with auranofin (1 µM) and erlotinib (ERL, 10 µM) for 48 h. Cell viability was analyzed by Crystal violet cell viability assays. One-way ANOVA followed by Tukey’s post-test, *n* = 3 independent biological replicates (mean ± SEM). **B** SUM159PT and MDA-MB-231 cells were treated with either auranofin (AF, 1 µM) and erlotinib (ERL, 10 µM) alone or in combination for 24 h. Expression of indicated proteins were analyzed by Western Blot analysis (*n* = 3 independent biological replicates). Vinculin was used as a loading control. Representative images of three independent experiments are shown. **C** SUM159PT and MDA-MB-231 cells were transfected with either scramble control or GSDME-specific siRNAs for 48 h. GSDME knockdown was confirmed by Western blot analysis. Vinculin was used as a loading control. Representative images of three independent experiments are shown (*n* = 3 independent biological replicates). **D** SUM159PT and MDA-MB-231 cells were transfected with either scramble control or GSDME-specific siRNAs for 24 h. Control and GSDME-depleted cells were treated with auranofin (AF, 1 µM) and erlotinib (ERL, 10 µM) combination for 48 h. Cell viability was analyzed by Crystal violet cell viability assays. One-way ANOVA followed by Tukey’s post-test, *n* = 3 independent biological replicates (mean ± SEM). **E** SUM159PT cells were pre-treated with ROA scavenger (NAC, 5 mM) for 3 h and subsequently treated with auranofin (AF, 1 µM) and erlotinib (ERL, 10 µM) for 24 h. Expression of indicated proteins was analyzed by Western blot analysis. Vinculin was used as a loading control. Representative images of three independent experiments are shown (*n* = 3 independent biological replicates).

To delineate the mechanism of cell death, TNBC cells were treated with auranofin and erlotinib either alone or in combination, in the presence of specific cell death inhibitors. Pretreatment with the pan-caspase inhibitor Q-VAD-FMK or the ferroptosis inhibitor ferrostatin-1 failed to rescue SUM159PT and MDA-MB-231 cells from auranofin–erlotinib combination-induced death (Fig. 5A). In contrast, the ROS scavenger N-acetylcysteine (NAC) [21, 24] significantly restored cell viability, suggesting that the cytotoxicity of the combination treatment is primarily mediated through oxidative stress (Fig. 5A).

Western blot analysis revealed that while erlotinib alone had no effect on gasdermin cleavage, auranofin monotherapy induced marked cleavage of gasdermin E (GSDME), but not gasdermin D (GSDMD). Notably, auranofin-erlotinib combination treatment led to more robust GSDME cleavage compared to auranofin treatment alone (Fig. 5B), suggesting synergistic cleavage of GSDME by combination treatment. GSDME cleavage is a recognized biochemical hallmark of pyroptosis in cancer cells and has been linked to the conversion of apoptotic signals into pyroptosis [25]. Notably, silencing GSDME expression significantly rescued SUM159PT and MDA-MB-231 cells from combination-treatment-induced cell death (Fig. 5C, D), confirming GSDME-dependent pyroptosis. Interestingly, NAC pretreatment prevented GSDME cleavage (Fig. 5E) and rescued cell death (Fig. 5A), whereas pan-caspase inhibitor Q-VAD-FMK failed to restore cell viability (Fig. 5A). indicating that oxidative stress acts upstream of GSDME activation. These findings indicate that oxidative stress lies upstream of GSDME activation and that auranofin–erlotinib induces a non-canonical, ROS-dependent GSDME cleavage independent of caspase activity. This observation is consistent with emerging evidence that reactive oxygen species can directly or indirectly activate pyroptotic pathways independent of the canonical caspase cascade [26]. Together, these results demonstrate that combined auranofin–EGFR inhibitor treatment elicits ROS-driven, caspase-independent GSDME-dependent pyroptosis in TNBC cells, revealing a previously unrecognized, redox-regulated mechanism of cell death that may be therapeutically exploited.

### TXNRD3 depletion or inhibition increases EGFR phosphorylation through a ROS-dependent mechanism in TNBC cells

The activating phosphorylation of EGFR at Y1068 has been associated with improved responses to EGFR inhibitors in lung cancer patients [27]. To evaluate EGFR activation status in TNBC, we examined EGFR expression and phosphorylation in a TNBC tissue microarray. Although a large proportion of TNBC tumors displayed strong total EGFR expression (localized to both the plasma membrane and cytoplasm), phosphorylated EGFR (p-EGFR, Y1068) was largely absent (Fig. S6). This lack of active EGFR may partly explain the poor response rates of TNBC patients to EGFR-targeted therapies despite high total EGFR expression.

Given the established role of redox signalling in EGFR activation, we investigated whether TXNRD3, a key redox-regulating enzyme, influences EGFR phosphorylation through modulation of intracellular oxidative balance. The EGFR kinase domain contains six cysteine residues, including Cys797 within the ATP-binding pocket. Oxidation of Cys797 has been reported to enhance EGFR kinase activity and autophosphorylation at Y1068 [28]. Since TXNRD3 maintains intracellular redox homeostasis by reducing oxidized thiols [17], we hypothesized that loss of TXNRD3 would increase ROS accumulation and promote EGFR oxidation and activation.

**Fig. 6:**
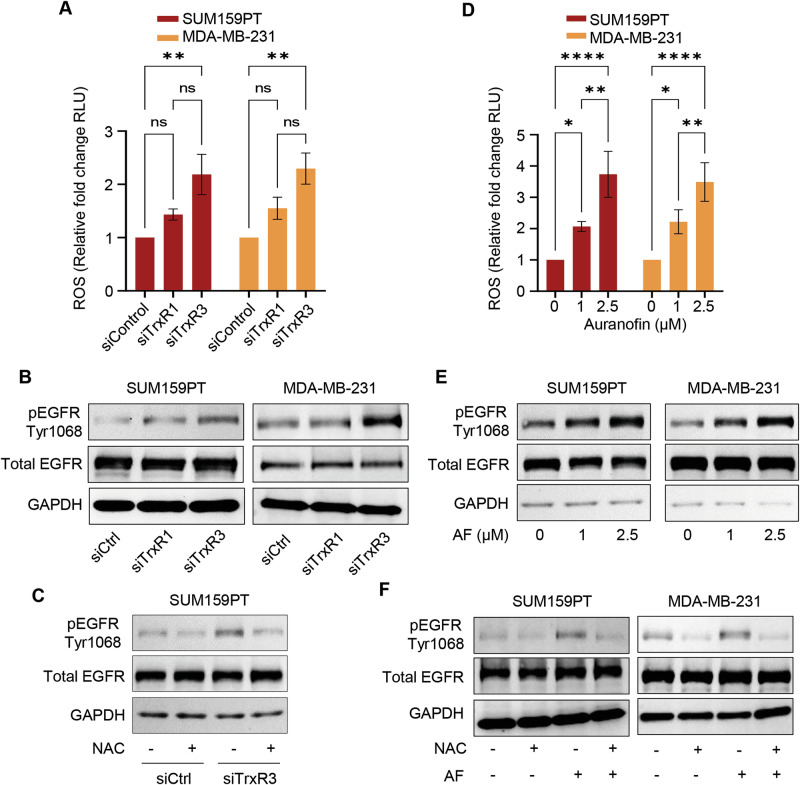
TXNRD3 depletion or inhibition increases EGFR phosphorylation in TNBC cells. **A** SUM159PT and MDA-MB-231 cells were transfected with either control siRNAs, TrxR1-specific siRNAs, or TrxR3-specific siRNAs for 48 h, and intracellular ROS levels were analyzed. One-way ANOVA followed by Tukey’s post-test, *n* = 3 independent biological replicates (mean ± SEM). **B** SUM159PT and MDA-MB-231 cells were transfected with TrxR1- or TrxR3-specific siRNAs as described above. Protein levels of phospho-EGFR (Y1068) and total EGFR were analyzed by Western blot analysis. Representative images of three independent experiments are shown (*n* = 3 independent biological replicates). GAPDH was used as a loading control. **C** SUM159PT and MDA-MB-231 cells were transfected with either TrxR1- or TrxR3-specific siRNAs for 16 h. Cells were then treated with 10 mM N-acetyl cysteine (NAC) for 36 h. Protein levels of phospho-EGFR (Y1068) and total EGFR were analyzed by Western blot analysis (*n* = 3 independent biological replicates). **D** SUM159PT and MDA-MB-231 cells were treated with auranofin (0–2.5 µM) for 24 h, and intracellular ROS levels were analyzed. One-way ANOVA followed by Tukey’s post-test, *n* = 3 (mean ± SEM). **E** SUM159PT and MDA-MB-231 cells were treated with auranofin (0–2.5 µM) for 24 h. Protein levels of phospho-EGFR (Y1068) and total EGFR were analyzed by Western blot analysis (*n* = 3 independent biological replicates). **F** SUM159PT and MDA-MB-231 cells were treated with 10 mM NAC for 3 h, followed by the treatment with auranofin (0–2.5 µM) for 24 h. Protein levels of phospho-EGFR (Y1068) and total EGFR were analyzed by Western blot analysis (*n* = 3 independent biological replicates).

Consistent with this hypothesis, siRNA-mediated TXNRD3 knockdown significantly elevated intracellular ROS levels in SUM159PT and MDA-MB-231 cells, whereas TXNRD1 knockdown had minimal effect (Fig. 6A). TXNRD3 depletion markedly increased EGFR phosphorylation at Y1068 in both cell lines (Fig. 6B), accompanied by enhanced ERK1/2 phosphorylation (Fig. S7A), indicating downstream pathway activation. In contrast, TXNRD1 depletion produced only marginal changes in EGFR or ERK1/2 phosphorylation. Pretreatment of TXNRD3-depleted cells with the ROS scavenger N-acetylcysteine (NAC for 16 h substantially reduced EGFR phosphorylation (Y1068) (Fig. 6C), confirming that the effect of TXNRD3 loss on EGFR activation is mediated by oxidative stress.

We next assessed whether pharmacological inhibition of TXNRD3 using auranofin recapitulates these findings. Auranofin treatment induced a concentration-dependent increase in intracellular ROS levels in both SUM159PT and MDA-MB-231 cells (Fig. 6D) and significantly enhanced EGFR phosphorylation at Y1068 (Fig. 6E). This increase was abolished by NAC pretreatment (Fig. 6F), establishing ROS dependence. Auranofin also elevated phosphorylation of downstream effectors ERK1/2 and AKT (Fig. S7B), consistent with EGFR pathway activation.

Collectively, these results demonstrate that TXNRD3 depletion or pharmacological inhibition promotes ROS accumulation, leading to oxidative activation of EGFR and its downstream signalling in TNBC cells. This redox-mediated EGFR activation provides a mechanistic basis for the enhanced sensitivity of TXNRD3-deficient TNBC cells to EGFR inhibitors.

### Auranofin-erlotinib combination treatment exerts significant anti-cancer activity in vivo

**Fig. 7:**
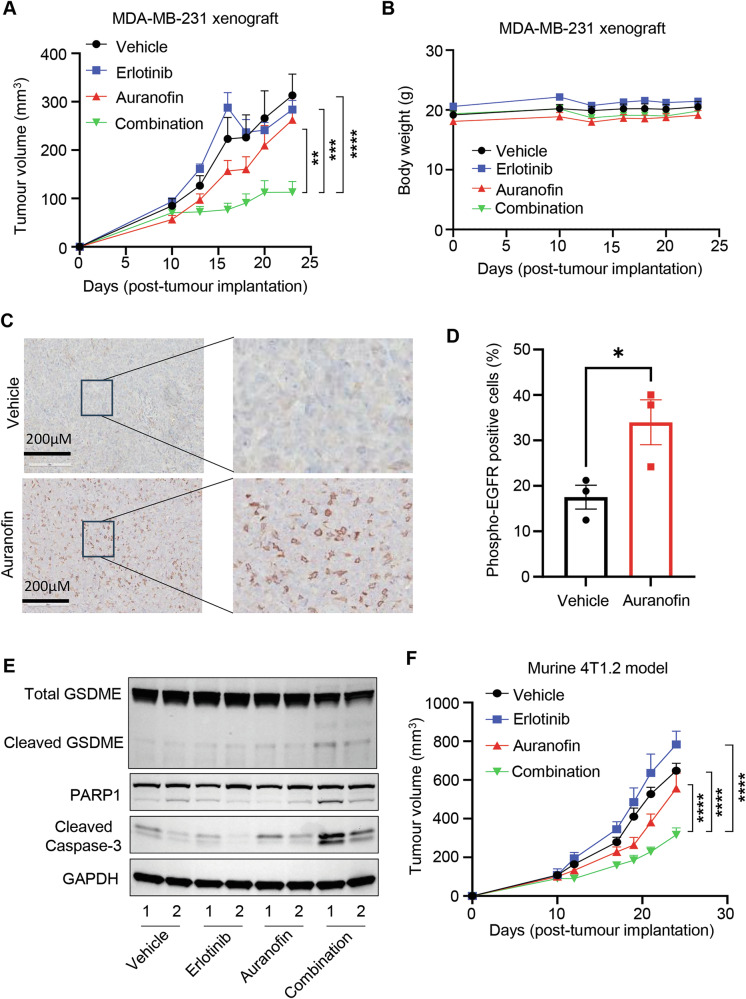
Auranofin-erlotinib combination treatment exerts significant anti-cancer activity in vivo. Tumor growth (**A**) and body weight (**B**) in Balb/c Nude mice orthotopically injected with MDA-MB-231 cells following treatment with vehicle, erlotinib (50 mg/kg, Monday-Friday, oral gavage), auranofin (5 mg/kg, Monday-Friday, ip), or combination for two weeks. Treatment started when the tumor reached 50-100 mm^3^. Data are presented as mean ± SEM (*n* = 6 mice/group); Two-way ANOVA followed by Tukey’s post-test was performed on tumor volumes, **p < 0.01, ***p < 0.001, ****p < 0.0001. Representative IHC images of phospho-EGFR (Y1068) staining of primary MDA-MB-231 tumors treated with vehicle or auranofin for 2 weeks (**C**). Quantification of phospho-EGFR-positive tumor cells in the primary MDA-MB-231 tumors. The percentage of phospho-EGFR-positive tumor cells is presented as mean ± SD (*n* = 3 tumors/group) (**D**). One-way ANOVA with Sidak’s multiple comparisons test, *p < 0.05, **p < 0.01. **E** Protein levels of cleaved GSDME, cleaved PARP1 and cleaved caspase-3 in MDA-MB-231 tumors treated with auranofin or erlotinib alone or in combination were analyzed by Western blot analysis. Two representative tumors were used for each treatment group. **F** Tumor growth in Balb/c mice orthotopically injected with murine 4T1.2 TNBC cells following treatment with vehicle, erlotinib (50 mg/kg, Monday-Friday, oral gavage), auranofin (5 mg/kg, Monday-Friday, ip), or combination for two weeks. Treatment started when the tumor reached 50–100 mm3. Data are presented as mean ± SEM (*n* = 6 mice/group); Two-way ANOVA followed by Tukey’s post-test was performed on tumor volumes, ****p < 0.0001.

We next evaluated the in vivo therapeutic efficacy of combined auranofin and erlotinib treatment using a human MDA-MB-231 xenograft model. Monotherapy with either auranofin (administered at half the maximum tolerated dose) or erlotinib did not significantly affect tumor growth compared with vehicle controls. In contrast, the combination of auranofin and erlotinib markedly suppressed MDA-MB-231 tumor growth in vivo (Fig. 7A), demonstrating a synergistic anti-tumor effect. The combination therapy was well-tolerated, with no significant differences in body weight between treatment and control groups (Fig. 7B).

To elucidate the mechanism underlying this enhanced efficacy, we examined EGFR activation in tumor tissues. Immunohistochemical analysis revealed that auranofin treatment significantly increased phosphorylation of EGFR at Y1068 (p-EGFR) in vivo (Fig. 7C). The proportion of phospho-EGFR-positive cells increased from 17.5 ± 3.2% in vehicle-treated tumors to 35.0 ± 4.1% following auranofin treatment, representing a mean difference of 16.5% ± 5.6% (Fig. 7D). These findings indicate that auranofin promotes EGFR activation in tumors, potentially enhancing their sensitivity to erlotinib.

We next assessed whether combination therapy induced pyroptosis in vivo. Consistent with in vitro results, Western blot analysis of tumor lysates showed that auranofin–erlotinib co-treatment markedly increased GSDME cleavage compared with either monotherapy or vehicle treatment (Fig. 7E). Concurrent increases in cleaved caspase-3 and PARP1 were also observed, supporting activation of pyroptotic and apoptotic signalling pathways in tumors.

To further confirm the therapeutic potential of this regimen, we tested the anti-cancer efficacy of auranofin-erlotinib combination therapy in a fully immunocompetent murine syngeneic model using 4T1.2 cells orthotopically implanted in the mammary fat pads of Balb/c mice. Similar to the xenograft model, monotherapy with either drug had minimal effect on tumor progression, whereas the combination treatment significantly reduced tumor growth (Fig. 7F).

Collectively, these data demonstrate that auranofin–erlotinib combination therapy exerts robust anti-tumor activity against TNBC in vivo, driven by auranofin-induced redox modulation, EGFR activation, and GSDME-mediated pyroptosis. These findings support this combination as a promising therapeutic strategy for EGFR inhibitor–refractory TNBC.

## Discussion

TNBCs are commonly characterized by EGFR overexpression [5, 6], which has led to several clinical trials evaluating the efficacy of EGFR-targeted therapies, including small-molecule EGFR inhibitors and anti-EGFR monoclonal antibodies. However, clinical outcomes have been disappointing, with minimal response rates and no significant survival benefit both as monotherapy and in combination with standard chemotherapy treatments. Phase II clinical trials of first-generation EGFR inhibitors, such as gefitinib and erlotinib monotherapy, demonstrated objective response rates of only 0–3% in patients with metastatic or recurrent breast cancer [10, 29]. Similarly, combining erlotinib with standard chemotherapy agents such as Bendamustine failed to show clinical efficacy in late-stage TNBC [30]. A second-generation EGFR inhibitor, afatinib, also failed to achieve measurable responses in metastatic TNBC patients [31].

Anti-EGFR monoclonal antibodies such as cetuximab have also yielded limited benefit [11, 32, 33]. Although cetuximab in combination with carboplatin showed slightly higher response rates (16%) compared to monotherapy (6%), the improvement was not statistically significant [11]. Other clinical trials combining cetuximab with cisplatin or ixabepilone also failed to demonstrate consistent benefit [32, 33]. Thus, despite EGFR overexpression, TNBCs remain largely refractory to EGFR-targeted therapy. Several mechanisms have been proposed for this intrinsic resistance, including compensatory activation of receptor tyrosine kinases such as MET, HER3, and AXL [34], though their therapeutic relevance remains to be fully validated. Understanding why EGFR-overexpressing TNBCs fail to respond to EGFR-targeted agents, therefore, remains a critical challenge.

To address this, we performed a genome-wide CRISPR/Cas9 knockout screen using the human GeCKO v2A [35, 36] library to identify genetic determinants of erlotinib resistance in TNBC. Our unbiased screen identified thioredoxin reductase 3 (TXNRD3), a mitochondrial redox enzyme, as a critical mediator of resistance to EGFR inhibitors in EGFR-high MDA-MB-231 cells. Functional validation confirmed that genetic depletion of TXNRD3, but not its cytosolic isoform TXNRD1, significantly sensitized TNBC cells to both first-generation (erlotinib) and third-generation (osimertinib) EGFR inhibitors. These findings align with previous studies implicating TXNRD3 in resistance to other kinase inhibitors, such as sorafenib, in leukemia cells [17].

Recent single-cell studies further underscore the importance of adaptive redox remodeling in driving EGFR inhibitor resistance in TNBC [37]. Notably, Pellecchia et al. employed single-cell lineage tracing to map adaptive resistance trajectories following EGFR inhibition and identified dynamic redox remodeling and IGFBP2-driven survival programs as key mediators of drug tolerance [37]. These findings are highly concordant with our genome-wide CRISPR/Cas9 screen, which independently identified a redox-dependent resistance mechanism mediated by TXNRD3 and flagged IGFBP family member IGFBP6 among resistance-associated candidates. Together, these complementary approaches, single-cell lineage tracing and functional genetic screening, converge on a model in which redox plasticity and growth factor–associated adaptive states enable TNBC cells to evade EGFR-targeted therapies.

The thioredoxin (Trx) and thioredoxin reductase (TXNRD) systems play central roles in maintaining intracellular redox homeostasis and are frequently upregulated in cancers, including TNBC [21, 24, 38], where they contribute to proliferation, survival, and drug resistance [39]. Amongst the three known isoforms of TXNRD, TXNRD1 is located in the cytosol, TXNRD2 in mitochondria [40], and TXNRD3 in both cytosol (66.6 kDa) and mitochondria (70.7 kDa) [17]. We previously reported that TXNRD1 inhibition using the FDA-approved gold compound auranofin suppresses TNBC growth in vitro and in vivo [21]. Notably, several studies have reported that TXNRD3 expression is significantly higher in TNBC tumor tissues compared to non-TNBC tumors and normal breast epithelia, suggesting a potential role for this enzyme in TNBC pathogenesis and therapy resistance [18]. In agreement with these reports, we found that TXNRD3, but not TXNRD1 expression, correlates with reduced EGFR inhibitor sensitivity in TNBC cells. Mechanistically, we observed a significantly higher accumulation of reactive oxygen species (ROS) upon depletion of TXNRD3 (the mitochondrial isoform) compared to TXNRD1. Furthermore, while TXNRD1 inhibition using the selective inhibitor TRi-1, [22, 23] effectively suppressed TXNRD1 redox activity, it did not enhance TNBC sensitivity to erlotinib or osimertinib, reinforcing the specific role of TXNRD3 in mediating resistance.

Auranofin, an FDA-approved thioredoxin reductase inhibitor that targets both TXNRD1 and TXNRD3 (19), effectively reduced TXNRD3 levels in TNBC cells. A sub-lethal dose of auranofin combined with either erlotinib or osimertinib produced synergistic anti-cancer effects in EGFR-high TNBC cell lines in vitro and significantly enhanced the anti-tumor efficacy of erlotinib in both MDA-MB-231 xenograft and immunocompetent 4T1.2 syngeneic TNBC models in vivo. These findings indicate that pharmacologic inhibition of TXNRD3 sensitizes TNBCs to EGFR inhibition, providing a clinically translatable therapeutic strategy.

Mechanistically, we sought to understand why TNBCs with high EGFR expression exhibit poor responses to EGFR inhibitors and how TXNRD3 inhibition alters this sensitivity. EGFR is traditionally localized at the plasma membrane, where it functions as a receptor tyrosine kinase driving mitogenic signaling [41]. However, recent evidence indicates that EGFR can also be localized in intracellular organelles, including the nucleus and cytosol [42, 43]. Increased intracellular EGFR has been linked to cetuximab resistance in both non–small-cell lung cancer [44, 45] and TNBCs [43], suggesting that mislocalization of EGFR may limit drug accessibility and contribute to therapeutic resistance.

Beyond localization, EGFR activity is modulated by redox-dependent mechanisms. Specifically, oxidation or sulfenylation of cysteine residues within the kinase domain in response to elevated intracellular ROS, particularly cysteine 797 [28], enhances receptor phosphorylation and activity. Consistent with these findings, we observed that both TXNRD3 depletion and auranofin treatment significantly increased intracellular ROS levels, leading to elevated phosphorylation of EGFR at Y1068 in SUM159PT and MDA-MB-231 cells. Importantly, pre-treatment of these cells with a ROS scavenger, N-acetylcysteine, attenuated this phosphorylation, confirming that TXNRD3 inhibition activates EGFR via a ROS-dependent mechanism. This redox-driven EGFR activation may restore the receptor’s “druggable” active conformation, enhancing its susceptibility to EGFR inhibitors.

Activated phosphorylated EGFR (Y1068) correlates with improved responses to erlotinib in non–small-cell lung cancer patients [27]. Therefore, redox modulation via TXNRD3 inhibition may represent a strategy to re-sensitize EGFR-overexpressing but inactive TNBCs to EGFR-targeted therapy.

In summary, our study identifies TXNRD3 as a previously unrecognized determinant of EGFR inhibitor resistance in TNBC. By linking redox homeostasis to EGFR activation status, we provide a mechanistic rationale for combining the redox-modulating agent auranofin with EGFR inhibitors to overcome intrinsic resistance in EGFR-high TNBCs. These findings highlight TXNRD3 as both a predictive biomarker and **a** therapeutic target, offering a promising direction for future clinical translation. Further studies are warranted to identify molecular markers of response and to stratify TNBC patients who are most likely to benefit from this combination strategy.

## Materials and methods

### Cell lines, reagents, and patient tissue samples

All breast cancer cell lines were obtained from the American Type Culture Collection (ATCC). The SUM159PT cell line was kindly provided by Prof Sunil Lakhani, UQCCR, Australia. The 4T1.2 cell line was kindly provided by Dr. Robin Anderson, Olivia Newton-John Cancer Research Institute, Australia. CRISPR/Cas9 EGFR knockout (KO) and CRISPR/Cas9 Vector control MDA-MB-231 cells were generated by Prof Chia-Hwa Lee (Taipei Medical University, Taiwan) [46]. All breast cancer cell lines were cultured in DMEM media supplemented with 10% fetal bovine serum (FBS) and 100 nM Sodium Selenite. All cell lines were tested for Mycoplasma infection and authenticated using short tandem repeat (STR) profiling by scientific services at QIMR Berghofer Medical Research Institute. Auranofin was purchased from Cayman Chemicals (Cat #: 15316). Erlotinib (Cat #: S7786) and osimertinib (Cat #: S7297) were purchased from Selleck Chemicals. TRi-1 was purchased from MedChem Express (Cat #: HY-125006. The list of antibodies used in this study is provided in Supplementary Table S1. The human TNBC patient tissue microarray was provided by the Mater Biobank.

### Genome-wide CRISPR/Cas9 knockout library screen

In this study, we used the Human GeCKOv2A & 2B CRISPR knockout pooled library to identify genes responsible for erlotinib resistance in TNBC cells. The library was a gift from Feng Zhang (Addgene # 1000000049) [47]. The workflow of this forward CRISPR-based genetic screen is illustrated in Fig. 1A. Firstly, we established a stable Cas9-expressing MDA-MB-231 cell line by lentiviral transduction of the Cas9 coding sequence. We then transduced Cas9-expressing MDA-MB-231 cells with GeCKO v2A and B library, which contains 123,411 unique sgRNA sequences targeting 19,052 human genes and 1864 miRNAs (6 sgRNAs per gene, 4 sgRNAs per miRNA, and 1000 non-targeting control sgRNAs) at an MOI of 0.3 to ensure each cell represents one sgRNA. The transduced cells were then selected with 1 µg/mL of puromycin for 7 days to generate a mutant cell line. The mutant cells were then treated with vehicle (DMSO) and erlotinib (10 µM) for 7 days, respectively. After 7 days of treatment, the residual cells were collected, and genomic DNA was isolated from each treatment group. The sgRNA sequences were amplified and subjected to parallel amplicon sequencing at Ramaciotti Centre for Genomics to identify sgRNAs lost in erlotinib-treated cells.

### CRISPR data analysis

#### Exploratory analysis of sgRNA counts

For the exploration analysis of sgRNA counts, we employed several visualization techniques using R packages. Pie charts were generated using the ‘scales‘ package (version 1.3.0) to provide an overview of the distribution of sgRNA counts. Volcano plots, depicting the fold change and statistical significance of differential expression, were created using the ‘Enhanced Volcano‘ package (version 1.20.0). Additionally, waterfall plots were generated using the ‘waterfalls‘ package (version 1.0.0) to visualize changes in sgRNA abundance across different conditions.

#### Differential abundance analysis

Differential abundance analysis was conducted using the ‘edgeR‘ Bioconductor package (version 4.0.12) [48, 49]. Initially, lowly expressed genes were filtered out based on the criteria of having a count lower than the median count per library for at least two samples. Subsequently, the counts were normalized using the upper-quartile method [50], implemented at the 0.99 quantile within the ‘normLibSizes‘ function.

Differential abundance of sgRNA counts between treatments was assessed using gene-wise likelihood ratio tests within the ‘edgeR‘ framework. To control for multiple testing, resulting *p*-values were adjusted using the false discovery rate (FDR) method of Benjamini and Hochberg [51].

### TXNRD3 ELISA assays

SUM159PT and MDA-MB-231 cells (5 × 10^5^ cell) were seeded in 60 mm dish overnight and treated with auranofin (0-2.5 µM) for 24 h. Then, cells were collected, and protein was extracted. TXNRD3 levels were analyzed in the protein lysates using Human Thioredoxin Reductase 3 (TXNRD3) ELISA Kit (abx384053, Abbexa, UK) as per the manufacturer’s instructions.

### Intracellular ROS assays

Breast cancer cell lines, SUM159PT and MDA-MB-231, were seeded overnight (5000 cells/well) in black-walled 96-well plates and were treated with or without auranofin for 24 h. The media containing the drug was then removed, cells were washed with 1X PBS and incubated with 5µM H2DCFDA for 30 min at 370 °C, and fluorescence was measured using a H4 Synergy H4 Multi Mode Plate Reader (Syn Biotek).

### In vivo animal models

Animal experiments were approved by the QIMR Berghofer Medical Research Institute Animal Ethics Committee (approval no. P3667). All experiments involving the use of animals were performed in accordance with the QIMR Berghofer Animal Experiment Regulations and experimental guidelines.

For the human MDA-MB-231 xenograft, 3 × 106 MDA-MB-231 cells were prepared in 50% growth factor-reduced Matrigel (BD, Biosciences, Bedford, USA)/PBS and injected into the right 4th inguinal mammary fat pad of 6-week-old female Balb/c Nude mice. For murine 4T1.2 syngeneic models, 1 × 10^5^ cells prepared in 1X PBS were injected into the right 4th inguinal mammary fat pad of 6-week-old female immunocompetent Balb/c mice. For all in vivo mouse models, once tumor size reached ~30–50 mm^3^, mice were randomized blindly into four treatment groups: (i) vehicle, (ii) erlotinib (50 mg/kg, Oral, Monday-Friday), (iii) Auranofin (half-MTD, 5 mg/kg, ip, Monday-Friday), and (iv) combination for 3 weeks. Tumor growth was measured thrice weekly using a digital caliper. To calculate the tumor volume, the following formula was used: tumor volume = [Lx W^2^]/2, where W = width of the tumor and L = length of the tumor. The maximal tumor size permitted by our ethics committee was 1000 mm^3^. For all animal experiments, the maximal tumor size was not exceeded by more than the approved size.

### Statistical analysis

For animal experiments, a pragmatic sample size of 5 or 6 mice per treatment group was chosen to allow adequate calculation of parameter estimates whilst being feasible [52]. No formal statistical power calculations were performed, but our sample sizes are similar to those reported in previous publications [53, 54]. No exclusion has been made in this study. All quantitative data are presented as mean ± standard error of the mean (SEM), unless otherwise stated. The number of biological replicates (n) is specified in the Fig. legends. We considered the biological replicates as independent experiments performed on different days using independently prepared cell cultures. For comparisons between two groups, statistical significance was assessed using Student’s *t* test. For comparisons involving more than two groups, one-way or two-way analysis of variance (ANOVA) was performed as appropriate, followed by Tukey’s or Sidak’s multiple-comparison post hoc test as indicated in each Fig. legend. The p-values indicated are *p < 0.05, **p < 0.01, ***p < 0.001, and ****p < 0.0001. Data was analyzed using GraphPad Prism 9 (GraphPad Software, CA, USA).

## Supplementary information

Supplementary file

Supplementary file

## Data Availability

The datasets used and/or analyzed during the current study are available from the corresponding author on reasonable request. The materials used in this study are available from the corresponding author upon request. The whole genome sequencing data associated with the CRISPR library screening experiment has been submitted to GEO under the metadata file name “240910-GEO-ERL-CRISPR-RNAseq.xlsx” and with an accession code GSE279690.
