# Supplementary material for: Redox regulation of EGFR activation by thioredoxin reductase 3 drives resistance to EGFR inhibitors in triple-negative breast cancer
Source: Cell Death Discov. 2026 May 19;12:295. doi: 10.1038/s41420-026-03157-0 (PMC13350899; doi:10.1038/s41420-026-03157-0)
Supplement: Supplementary file 1 — Supplementary file [file 41420_2026_3157_MOESM1_ESM.docx]

**SUPPLEMENTARY METHODS:**

**Cell viability assays:**

EGFR-high breast cancer cells (SUM159PT, MDA-MB-231, HCC1806, and HCC1143) were seeded at the density of 3000 cells/well onto a white-walled clear bottom 96-wells plate overnight. Cells were then treated with auranofin and EGFR inhibitors, erlotinib or osimertinib, alone or in combination for 3 days, and cell viability was analyzed using the Real-Time-Glo™ MT Cell Viability Assay (Promega) as per the manufacturer’s guidelines. Synergy scores between different drug combination was calculated using Synergy Finder (1). To analyze the effect of auranofin and erlotinib combination therapy on EGFR-low breast cancer cells, MCF7, BT-474, and SKBR3 cells were seeded at the density of 3000 cells/well onto a clear 96-wells plate. Cells were treated with auranofin and erlotinib, alone or in combination, for 3 days, and cell growth was analyzed by MTS assays (Promega).

**siRNA transfections:**

SUM159PT and MDA-MB-231 cells were transfected with 20 nM of non-specific scramble small interfering RNAs (siRNAs), human TrxR1-specific, human TrxR3-specific, human EGFR-specific siRNAs, or human siGSDME-specific siRNAs using Lipofectamine RNAi MAX (Invitrogen Cat #: 13778030) per manufacturer’s instructions. The sequences of siRNAs used in manuscript are provided in supplementary table S2.

**Thioredoxin reductase 1 activity assays:**

Breast cancer cells were treated with auranofin for 24 h, then lysed, and cellular TrxR1 redox activity was measured by the DTNB reduction assay as described previously (2).

**Reverse Transcription-qPCR:**

SUM159PT and MDA-MB-231 cells were transfected with either scramble siRNA (siControl), TrxR1-specific siRNA, or TrxR3-specific siRNA for 48 hours. TrxR1 and TrxR3 gene expression analysis using reverse-transcriptase quantitative PCR (RT-qPCR) was performed using the Viia7 real-time PCR system (Applied Biosystems, US).

**Western blot:**

Breast cancer cells were either transfected with specific siRNAs as described above, or treated with auranofin (0-2.5 µM, 24 hours). Proteins were extracted using a 7M Urea buffer. Immunoblotting was performed as described previously (3) with the antibodies listed in Table S1. The Super Signal chemiluminescent ECL-plus (Amersham) was applied for antibody detection.

**Bioinformatics analysis:**

To obtain sensitivity to EGFR inhibitors, we downloaded the primary screen log fold change data ([primary-screen-logfold-change.csv](https://ndownloader.figshare.com/files/20237703)) from the PRISM Repurposing dataset (<https://depmap.org/repurposing/>) across CCLE breast cancer cell lines (we focused on EGFR inhibitors: dacomitinib, erlotinib, gefitinib, and osimertinib).

The gene expression data across CCLE breast cancer cell lines was also obtained from the DepMap portal (4) (https://depmap.org/portal/data_page/?tab=currentRelease; OmicsExpressionTPMLogp1HumanProteinCodingGenes.csv).

To assess ROS, we used the gene sets described in (5) (suppl table 5, gene sets HALLMARK_REACTIVE_OXYGEN_SPECIES_PATHWAY). For each gene set, a signature was obtained per cell line using the GSVA (Gene Set Variation Analysis (6), approach as implemented in R Bioconductor package GSVA.

The resulting combined data includes sensitivity to 4 EGFR inhibitors (dacomitinib, erlotinib, gefitinib, and osimertinib), the transcript per million counts for TXNRD3, and a gene signature value for ROS across 26 breast cancer cell lines from the CCLE, of which 12 are TNBC lines.

The linear regression comparing the sensitivity of each EGFR inhibitor to the ROS signature values across TNBC was implemented using the R base function “lm”, namely “lm (logfc ~ ROS)”. This provided the estimate of the regression slope for each compound. When plotting the relationship the inbuild ggplot function “geom_smooth (method=’lm’)” was used.

To calculate the Pearson correlation between sensitivity of each EGFR inhibitor to the ROS signatures and expression values, the base R function “cor” was used with default parameters. These correlations were calculated for all breast cancer cell lines, as well as per subtype, namely TNBC. For the breast cancer subtype annotation, we used the secondary tissue annotation column provided by DepMap in its cell line annotation file.

To explore the transcriptional changes induced by EGFRi resistance, we used the data described in (7) and shared in the gene Expression Omnibus GEO (Project id GSE189257) as raw counts. The data contains the transcriptional output for 6 samples, 3 parental and 3 resistant samples. The expression raw counts were normalized using the function calcNormFactors from the R Bioconductor package edgeR. The log CPM expression values were obtained after further transformation with the function CPM with parameter log=TRUE and prior. Count = 1 also from edgeR. These log CPM values were used to calculate gene signature values for each sample using GSVA as above.

**REFERENCES:**

1. Zheng S, Wang W, Aldahdooh J, Malyutina A, Shadbahr T, Tanoli Z*, et al.* SynergyFinder Plus: Toward Better Interpretation and Annotation of Drug Combination Screening Datasets. Genomics Proteomics Bioinformatics **2022**;20:587-96

2. Raninga PV, Di Trapani G, Vuckovic S, Tonissen KF. TrxR1 inhibition overcomes both hypoxia-induced and acquired bortezomib resistance in multiple myeloma through NF-small ka, Cyrillicbeta inhibition. Cell Cycle **2016**;15:559-72

3. Raninga PV, Lee A, Sinha D, Dong LF, Datta KK, Lu X*, et al.* Marizomib suppresses triple-negative breast cancer via proteasome and oxidative phosphorylation inhibition. Theranostics **2020**;10:5259-75

4. Arafeh R, Shibue T, Dempster JM, Hahn WC, Vazquez F. The present and future of the Cancer Dependency Map. Nat Rev Cancer **2025**;25:59-73

5. Oren Y, Tsabar M, Cuoco MS, Amir-Zilberstein L, Cabanos HF, Hutter JC*, et al.* Cycling cancer persister cells arise from lineages with distinct programs. Nature **2021**;596:576-82

6. Hanzelmann S, Castelo R, Guinney J. GSVA: gene set variation analysis for microarray and RNA-seq data. BMC Bioinformatics **2013**;14:7

7. Rashid NS, Boyd DC, Olex AL, Grible JM, Duong AK, Alzubi MA*, et al.* Transcriptomic changes underlying EGFR inhibitor resistance in human and mouse models of basal-like breast cancer. Sci Rep **2022**;12:21248

**SUPPLEMENTARY MATERIALS:**

**Table S1: List of antibodies.**

| **Marker** | **Cat #** | **Titration** | **Vendor** |
| --- | --- | --- | --- |
| TrxR1 | MAB7428 | 1:1000 | R&D Systems |
| TrxR3 | abx239135 | 1:1000 | Abbexa |
| Phospho-EGFR (Y1068) | 2234 | 1:1000 | Cell Signaling |
| Total EGFR | 4267 | 1:1000 | Cell Signaling |
| Vinculin | 13901 | 1:1000 | Cell Signaling |
| Phospho-ERK1/2 | 4370 | 1:1000 | Cell Signaling |
| Total ERK1/2 | 4695 | 1:1000 | Cell Signaling |
| Phospho-AKT1 | 4060 | 1:1000 | Cell Signaling |
| Total AKT1 | 9272 | 1:1000 | Cell Signaling |
| PARP | 9542 | 1:1000 | Cell Signaling |
| Cleaved caspase-3 | 9661 | 1:500 | Cell Signaling |
| GAPDH | RDS2275PC100 | 1:1000 | R&D Systems |

**Table S2: List of siRNAs.**

| **Gene** | **siRNA Sequence (Sense)** | **siRNA Sequence (Anti-sense)** |
| --- | --- | --- |
| EGFR | GGAUAUUCUGAAAACCGUAAAGGAA | GACCUAUAAGACUUUUGGCAUUUCCUU |
| TXNRD1 | ACAAGUACAUCUGCGAUCAACUCTA | UAGAGUUGAUCGCAGAUGUACUUGUUU |
| TXNRD3 | UGAUAACCUUGAAGCUAUUCUCCCC | CCACUAUUGGAACUUCGAUAAGAGGGG |
| siGSDME | GCUUCAGUGAACAUACUAAUUCUAT | UACGAAGUCACUUGUAUGAUUAAGAUA |

**SUPPLEMENTARY FIGURES:**

**Figure S1:**


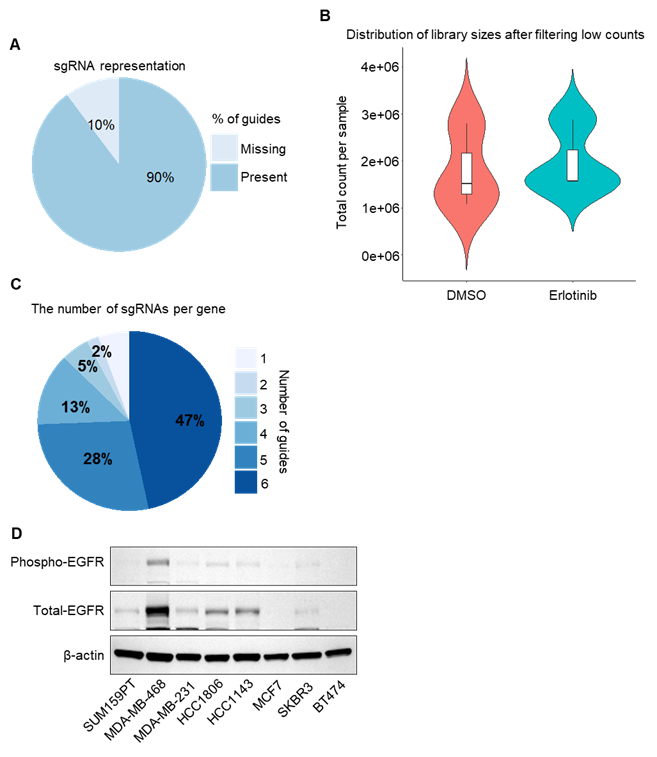


**Figure S1: Genome wide CRISPR screening using GeCKO v2 library in MDA-MB-231 cells.**

1. The pie chart showing the percentage of sgRNAs missing and present in MDA-MB-231 cells after transduction of the GeCKO v2 library and puromycin selection.
2. The pie chart representing the number of guides per gene. The chart also displays the percentage of genes targeted by a specific number of sgRNAs in our genome wide CRISPR screening using GeCKO v2 library.
3. The violin plot of the distribution of GeCKO v2 library. The figure illustrates the distribution of the sgRNA abundances in each library depicting total counts per condition in cells treated with DMSO and Erlotinib. These counts reflect the filtering of non-targeting sgRNAs and sgRNAs with low expression.
4. Expression of phosphorylated EGFR (Y1068) and total EGFR protein was analysed in a panel of breast cancer cell line using Western blot analysis. β-actin was used as a loading control.

**Figure S2:**


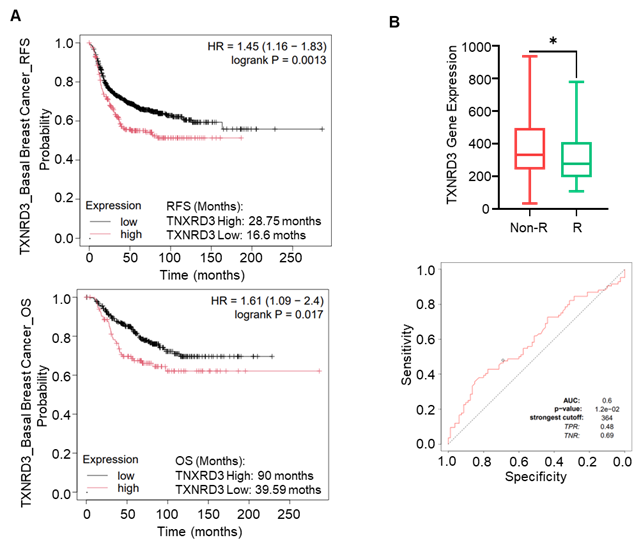


**Figure S2: Higher TXNRD3 expression correlates with poor prognosis and therapy response in TNBC patients.**

1. Effect of TXNRD3 gene expression on relapsed free survival (RFS) and overall survival (OS) of TNBC patients was analyzed by the Kaplan-Meier (KM) plotter.
2. Expression of TXNRD3 gene in chemotherapy responders and non-responders TNBC patients was analyzed using the ROC Plotter module of the KM plotter platform.
3. Area under the curve (AUC) was analyzed for the expression of TXNRD3 and its correlation with chemotherapy response in TNBC patients using the ROX Plotter module of the KM plotter platform.

**Figure S3:**


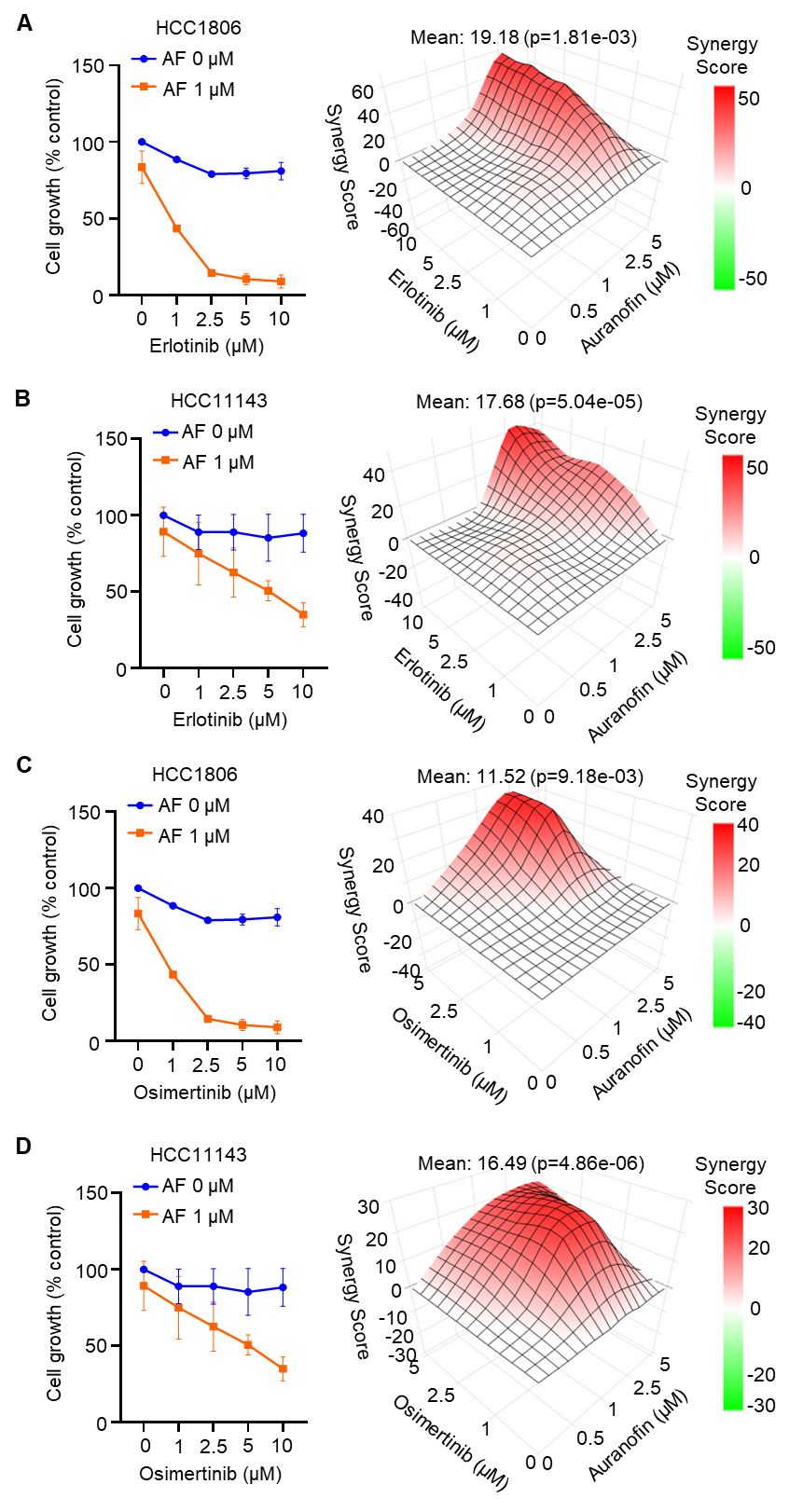


**Figure S3:** **Pharmacological inhibition of TXNRD3 using auranofin sensitized TNBC cells to EGFR inhibitors *in vitro*.**

**(A, B)** HCC1806 (A) and HCC1143 (B) cells were treated with auranofin (AF) (0-5 µM) and Erlotinib (0-10 µM), both alone and in combination, for 72 hours, and cell viability was analyzed by MT cell viability assays. For cell growth curves (left panel), results with only 1 µM AF are shown. Synergy score was calculated using Synergy Finder. One-way ANOVA followed by Tukey’s post-test, n = 3 (mean ± SEM).

**(C, D)** HCC1806 (C) and HCC1143 (D) cells were treated with auranofin (0-5 µM) and Osimertinib (0-10 µM), both alone and in combination, for 72 hours, and cell viability was analyzed by MT cell viability assays. For cell growth curves (left panel), results with only 1 µM AF are shown. Synergy score was calculated using Synergy Finder. One-way ANOVA followed by Tukey’s post-test, n = 3 (mean ± SEM).

**Figure S4:**


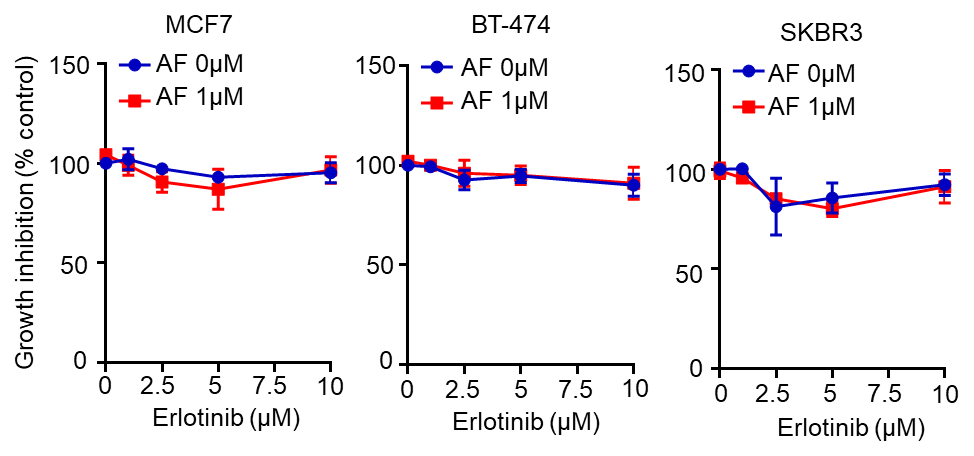


**Figure S4: Effect of auranofin on sensitizing EGFR-low breast cancer cells to erlotinib *in vitro*.**

EGFR-low non-TNBC cell lines including MCF7, BT-474, and SKBR3 were treated erlotinib (0-10 µM) alone or in combination with auranofin (AF) (1 µM) for 72 hours. Cell viability was analyzed by MT cell viability assay. One-way ANOVA followed by Tukey’s post-test, n = 3 (mean ± SEM).

**Figure S5:**


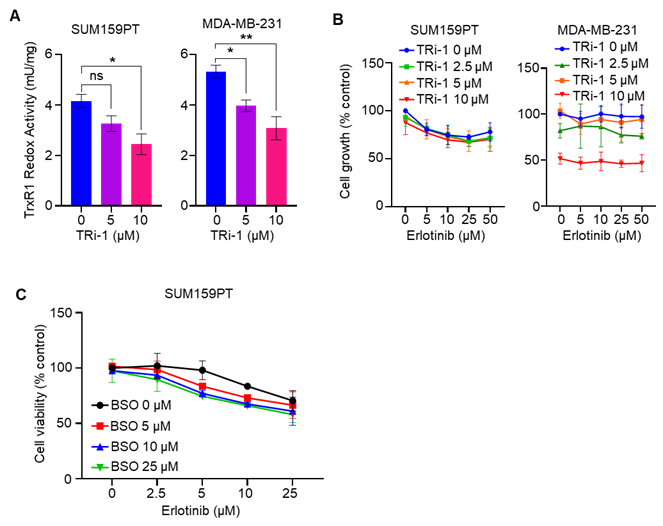


**Figure S5: Effect of thioredoxin reductase 1 specific inhibitor, TRi-1, on sensitizing TNBC cells to Erlotinib.**

1. SUM159PT and MDA-MB-231 cells were treated with TRi-1 (0-10 µM) for 24 hours. The redox activity of thioredoxin reductase 1 (TrxR1) was analyzed by the DTNB reduction assays. One-way ANOVA followed by Tukey’s post-test, n = 3 (mean ± SEM).
2. SUM159PT and MDA-MB-231 cells were treated with TRi-1 (0-10 µM) alone or in combination with erlotinib (0-50 µM) for 72 hours. Cell viability was analyzed by MT cell viability assay. One-way ANOVA followed by Tukey’s post-test, n = 3 (mean ± SEM).
3. SUM159PT cells were treated with glutathione inhibitor, BSO (0-25 µM) alone or in combination with erlotinib (0-25 µM) for 72 hours. Cell viability was analyzed by MT cell viability assay. Two-way ANOVA followed by Tukey’s post-test, n = 2 (two biological replicates, mean ± SD).

**Figure S6:**


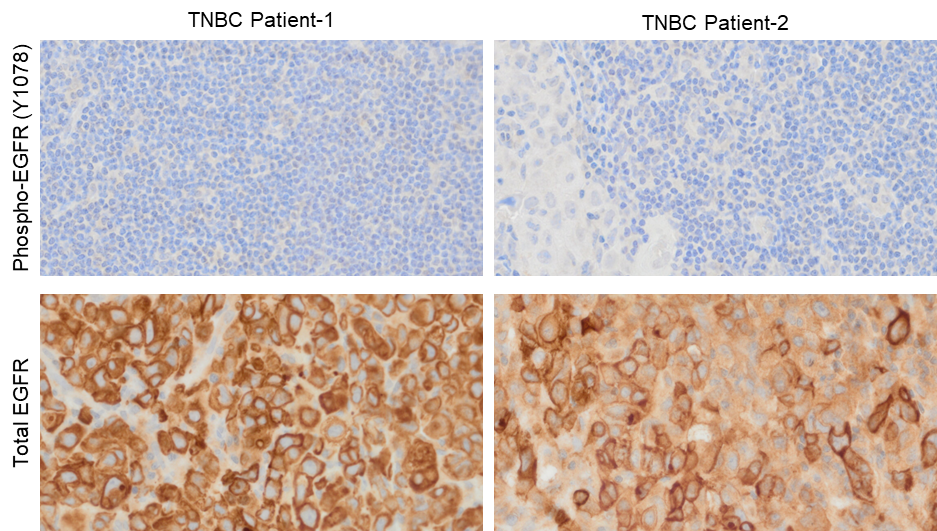


**Figure S6: Expression of Phospho-EGFR and total EGFR protein on TNBC patient tumour cells.**

Tissue microarray analysis of primary and treatment naïve TNBC patient tumours for the expression of phosphorylated EGFR (Y1078) protein and total EGFR protein. Representative images from two TNBC patient tumours are shown.

**Figure S7:**


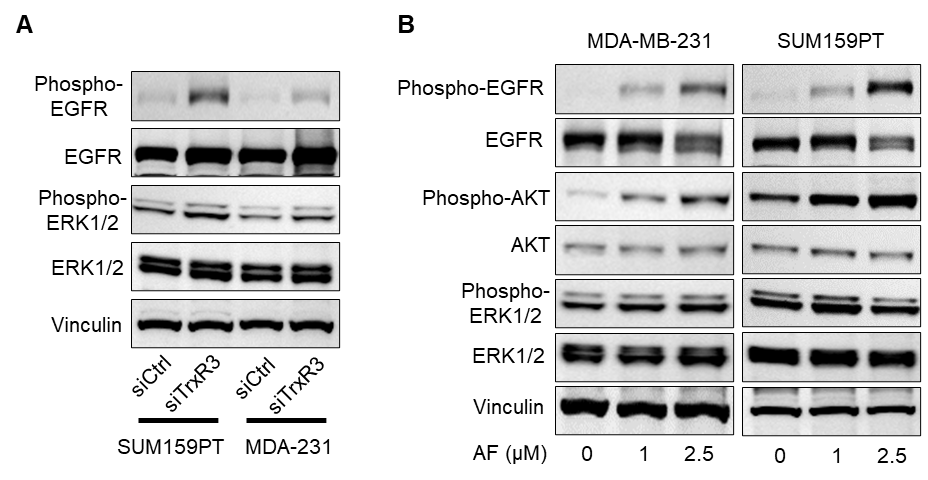


**Figure S7: TXNRD3 knockdown and inhibition activates EGFR signalling pathway in TNBC cells.**

1. SUM159PT and MDA-MB-231 cells were transfected with either control siRNAs, TrxR1-specific siRNAs, or TrxR3-specific siRNAs for 48 hours. Protein levels of phospho-ERK1/2 and total ERK1/2 were analyzed by Western blot analysis. Representative images of three independent experiments are shown. Vinculin was used as a loading control.
2. SUM159PT and MDA-MB-231 cells were treated with auranofin (0-2.5 µM) for 24 hours. Protein levels of phospho-ERK1/2, total ERK1/2, phospho-AKT, and total AKT were analyzed by Western blot analysis. Representative images of three independent experiments are shown. Vinculin was used as a loading control.
