# Supplementary material for: Redox regulation of EGFR activation by thioredoxin reductase 3 drives resistance to EGFR inhibitors in triple-negative breast cancer
Source: Cell Death Discov. 2026 May 19;12:295. doi: 10.1038/s41420-026-03157-0 (PMC13350899; doi:10.1038/s41420-026-03157-0)
Supplement: Supplementary file 2 — Supplementary file [file 41420_2026_3157_MOESM2_ESM.docx]

**Figure 1C**

**
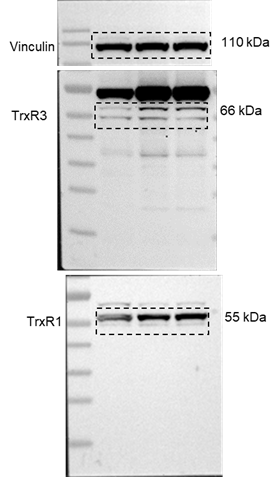
**

**Figure S1D**

**
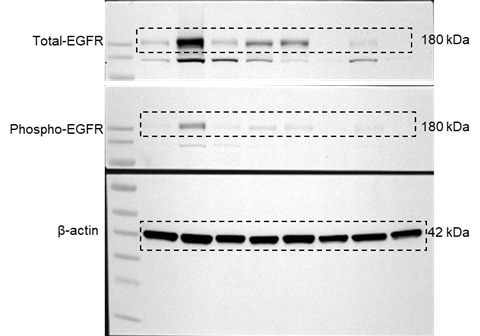
**

**Figure 4A**

**
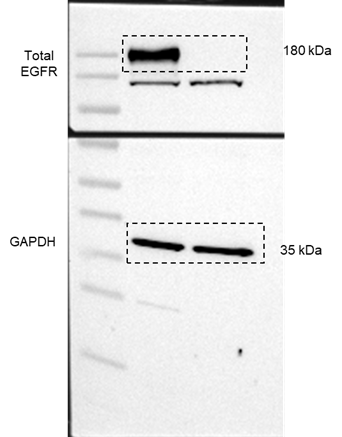
**

**Figure 4D**

**
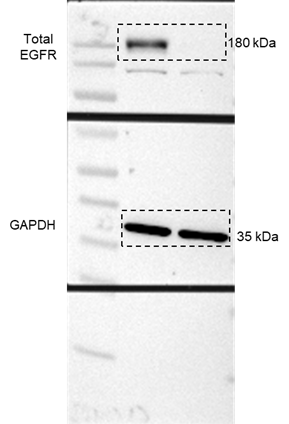
**

**Figure 5B**

**
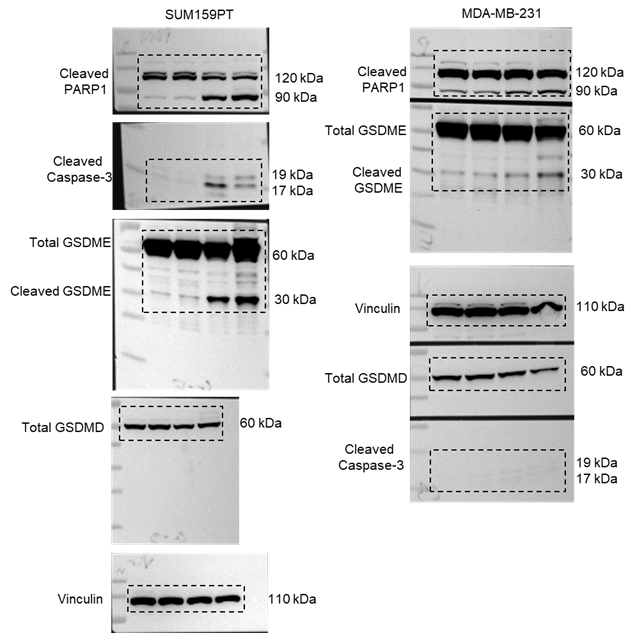
**

**Figure 5C**

**
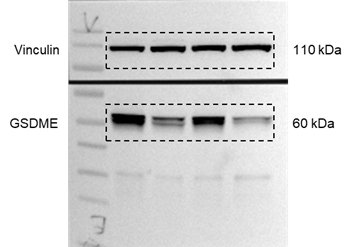
**

**Figure 5E**

**
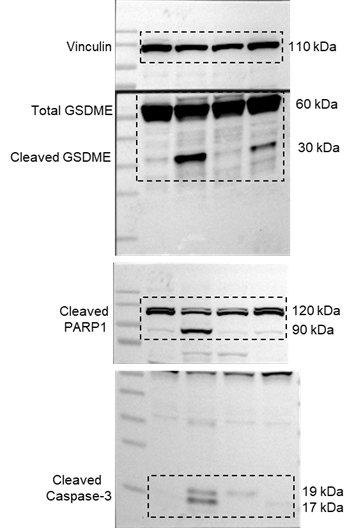
**

**Figure 6B**

**
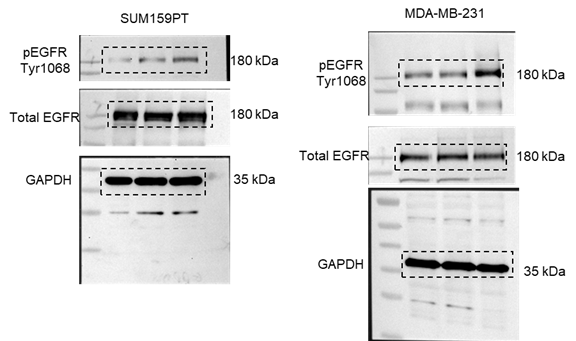
**

**Figure 6C**

**
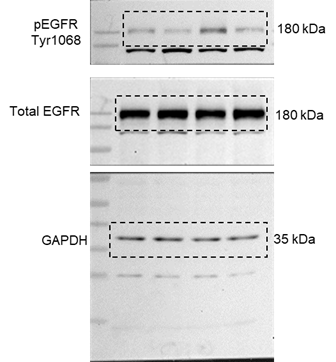
**

**Figure 6E**

**
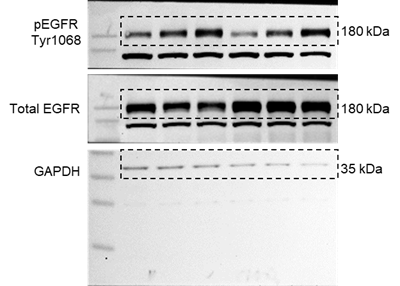
**

**Figure 6F**

**
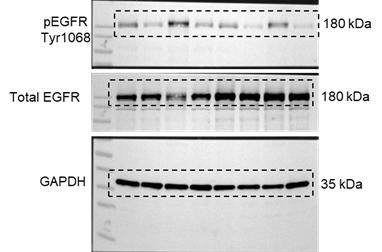
**

**Figure S6A**

**
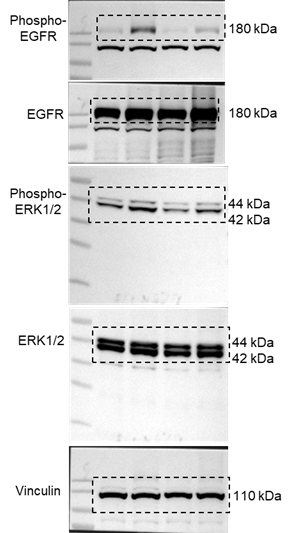
**

**Figure S6B**

**
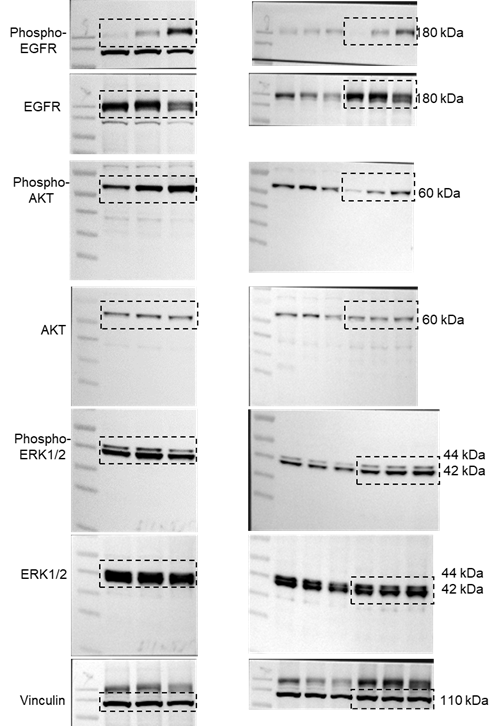
**

**Figure 7E**

**
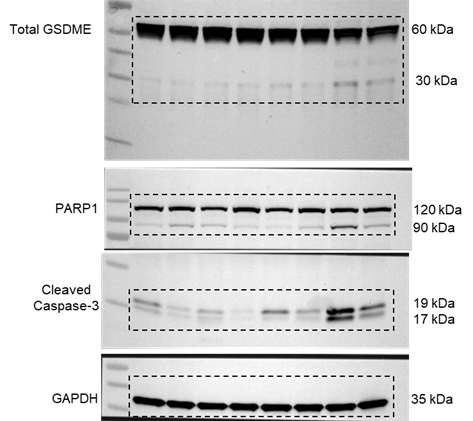
**
